# Demystifying a buzzword: Use of the term “human-animal-interface” in One Health oriented research based on a literature review and expert interviews

**DOI:** 10.1016/j.onehlt.2023.100560

**Published:** 2023-05-11

**Authors:** Sylvia Dreyer, Maren Dreier, Klaas Dietze

**Affiliations:** a Friedrich- Loeffler-Institut, Institute of International Animal Health/One Health, Greifswald, Germany; b Hannover Medical School, Institute for Epidemiology, Social Medicine and Health Systems Research, Hannover, Germany

**Keywords:** Human, Animal, Environment, Interface, One Health

## Abstract

As of today, 75% of infectious human diseases are caused by zoonotic pathogens, which use the interface between humans and animal species to cross. Due to this ability, zoonoses affect more than just one health sector and the effective control is a matter of the One Health concept. One defining feature of this concept is the “human-animal-interface”. However, even though the term is ubiquitously used in the field of infectious disease research, a clear definition of the term is lacking, leading to a rather nebulous understanding of what this interface really encompasses. Based on this observation, this study aimed to analyze the use of the term “human-animal-interface” in scientific literature to identify patterns and categories facilitating a scientific categorization.

A systematic literature search of two electronic databases was performed complemented by interviews with health experts in the field of zoonoses/One Health conducted between March 2019 and May 2021. From identified publications, keywords and interface descriptions were extracted and categorized. Interviews followed a questioning route, were audio recorded, transcribed, and qualitative content was inductively categorized.

Findings are based on 208 publications and 27 expert interviews. “Transmission” and “zoonosis” were the most frequent literature-based keywords, while the interviewees clearly favored “interface” followed by “contact”. Seven categories of contact interfaces were inductively derived: direct contact (physical contact), consumption of animal products, use of animal products (blood transfusion, skin), contact with animal products (blood, secretion, meat), indirect contact (dust, inhalation, droplets), environmental contact (same surface or food), vector contact). Precise descriptions of the interfaces varied greatly depending on the pathogen domain (bacterial, viral, fungal). Specific patterns could be identified that were consistent between the literature and experts.

The study results showed a general concordance in defining and describing the human-animal-interface indicating a general understanding of the term. However, studies on a larger scale are recommended (e.g. systematic review) to allow a more thorough view of the understanding and definition of the human-animal-interface.

## 1 Introduction

Zoonoses are diseases, which are transmissible between humans and animals. As of today, 75% of infectious human diseases are caused by zoonotic pathogens [1], which, for some of those pathogens (e.g. herpesviruses), lead back to the separation of the human from the simian line that goes along with host-pathogen co-evolution processes [2,3]. Throughout human history, several human behavior-associated factors seem to have influenced the frequency and intensity of zoonotic spillover events, such as a change in diet or habitat as well as the domestication of wild animal species and higher mobility [2,4]. Especially the destruction of natural areas due to the establishment of permanent settlements resulting in population growth of both, humans and domesticated animals, provided a good opportunity for pathogen spillover and the spread of diseases between species [2,5].

Efforts to tackle challenges of human, animal, and environmental health in a holistic approach have come together under the “One Health” concept, an approach that developed over roughly 2500 years starting in 500 BCE with the first Greek philosophers [6]. However, “One Health” was first born in 2003 [7] and officially used in 2004 at the “One World, One Health” symposium [8,9]. All relevant international organizations have acknowledged the importance of the approach and provide a definition on the meaning of it, such as the One Health High-Level Panel (OHHLEP) [10]. The panel was established in 2021 and defines One Health as an “integrated, unifying approach that aims to sustainably balance and optimize the health of people, animals, and ecosystems. It recognizes the health of humans, domestic and wild animals, plants, and the wider environment (including ecosystems) are closely linked and interdependent” [11].

The human-animal-interface plays a key role in the One Health approach. Especially in the light of the growing awareness of the One Health approach, the term human-animal-interface is broadly used throughout the literature. However, in contrast to One Health, there is no definition available for human-animal-interface. We postulate that due to the lack of a definition, the understanding of the human-animal-interface term varies greatly. To address this variation, this study aims to identify patterns and categories in the use of the term, thereby contributing to a scientific categorization.

## 2 Material and methods

This study includes a literature search and expert interviews for data collection. Main data were acquired by a systematic literature search.

### 2.1 Systematic literature search

The methods of the literature search were described according to the Preferred Reporting Items for Systematic reviews and Meta-Analyses (PRISMA) statement [12]. Studies were identified by systematic search in the bibliographic databases PubMed and ISI Web of Science. The search strategy combined search strings listed in the Supplementary Data. No filters (e.g. year or type of article) were set on purpose. The last update was run in 2022 on September 1st. After excluding duplicates, the records were screened for eligibility by the following criteria: (a) interface had to be mentioned, (b) transmission pathways had to be described and (c) the described transmission pathway had to be linked to a specific pathogen.

From identified publications, keywords and interface descriptions were extracted and categorized inductively (see Table 1, Table 2). Frequencies of identified keywords and interface descriptions were reported in absolute numbers and percentages (more than one mention was counted only once per study). The search, selection, and analysis of studies were performed by SD.

**Table 1:**
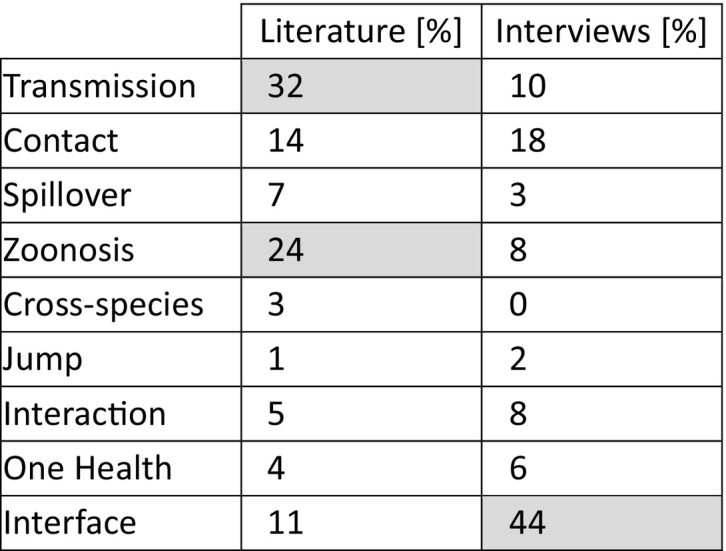
Relative frequencies of keywords to describe the human-animal-interface based on literature review and experts' interviews.

Literature (n = 208) and interview (*n* = 27) data were analyzed for the relative frequency of used synonyms to describe or identify a spillover event. The total number of entries was summed up and their relative frequency was calculated and is shown in %. Relative frequencies higher than 20% are shaded in grey.

**Table 2:**
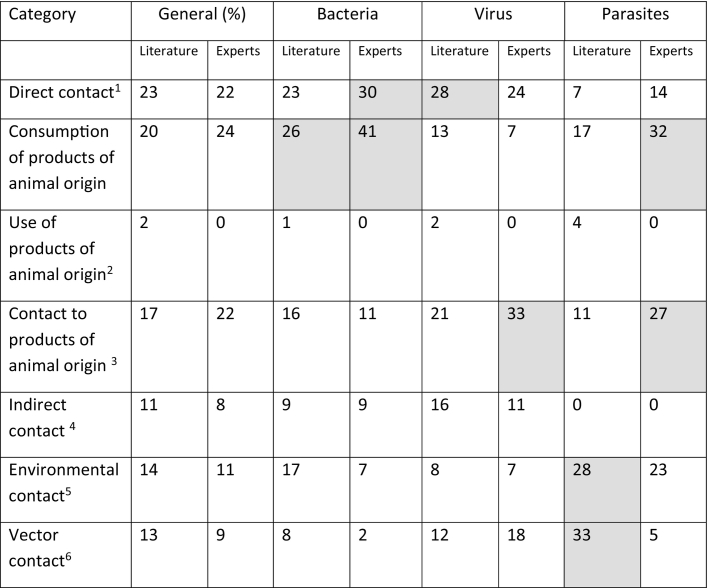
Relative frequencies of contact interface categories in different pathogen domains based on literature review and experts' interviews.

Results were analyzed according to their described way of pathogen-specific spillover. Seven categories of contact interfaces were identified and analyzed depending on their relative frequency of mentioning. Subsequently, contact interfaces were analyzed in general and pathogen domain-specific (bacteria, virus, parasite). Legend: ^1^physical contact, ^2^blood transfusion, skin from animals, ^3^blood, secretion, meat, ^4^dust, inhalation, droplets, ^5^same surface, same food. Relative frequencies higher than 24% are shaded in grey. Fungal spillover was mentioned as direct contact by one expert only and therefore not included in the table.

### 2.2 Expert interviews

The reporting followed the Consolidated criteria for reporting qualitative research (COREQ) checklist [13]. National and international participants were selected purposively according to their expertise on zoonoses/One Health. At the time of the interviews, all participants were actively working in their field of expertise and there were no restrictions regarding sex, age, or native language. Participation was voluntary and obtained by written consent. The interviews were approved by the ethics committee of the University of Greifswald (registration number BB 139/21).

Forty-four experts working in the field of zoonoses/One Health were contacted using an already existing network based on contacts to current and former colleagues at the Friedrich-Loeffler-Institut, the Food and Agriculture Organization of the United Nations (FAO), the World Health Organization, the World Organisation for Animal Health, the United States Centres for Disease Control and the German Federal Ministry for Economic Cooperation and Development. In total, 27 interviews were conducted (response 27/44; 61.4%). The interviews took place face-to-face (*n* = 12) or via Zoom video calls (*n* = 15) performed by SD. The interviews were conducted during two time periods in March – May 2019 (n = 12) and March – May 2021 (n = 15). For further information see Supplementary Data.

The interviews included 5 open-ended questions (see Table S2). Questions were asked only once and ad hoc and not shared beforehand. The questions were pilot tested with the first two participants, and as no changes were made, these 2 interviews were included in the data set. The Zoom interviews were audio recorded and transcribed. The transcripts were returned to the participants for proofreading, validation, and signature. All participants validated their version. Interview data were analyzed according to Mayring [14]. Variations in theme by age, gender, experience, and qualifications were not examined. Participants' quotations were included anonymously. Transcription and analysis of the interviews were performed by SD.

## 3 Results

### 3.1 Identification of relevant literature records over time

**Fig. 1:**
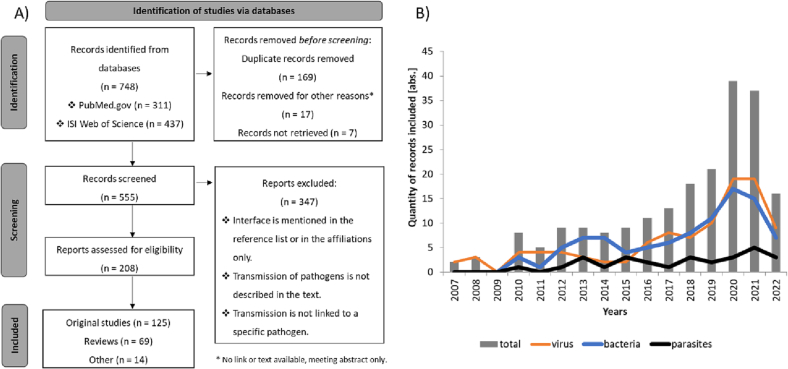
**Identification of literature hits.** A) PRISMA-S [12] flow diagram to identify literature hits. Adapted from McKenzie et al. and Scheer et al. B) Based on total records (grey columns, *n* = 208), pathogen-specific records were extracted for virus-specific (orange line), bacteria-specific (blue line), and parasite-specific (black line) literature. The absolute quantity of records is shown over time starting with first relevant appearance thereof. (For interpretation of the references to colour in this figure legend, the reader is referred to the web version of this article.)

The process of record selection is shown in Fig. 1A. 208 out of 748 literature hits were considered relevant and included in the study. The results show first matches in 2007 with increasing quantity over time, a peak in 2020 followed by a slight decrease in 2021 and a stronger decrease in 2022. General findings were grouped according to their pathogen domain affiliation permitting a domain-specific assessment of the records over time. For literature results with viral or bacterial pathogens, data showed an increase over time until 2020 with a slight decrease in 2021, whereas literature with parasitic content remained at a low level (see Fig. 1B).

### 3.2 Identification of pathogen domains and species

**Fig. 2:**
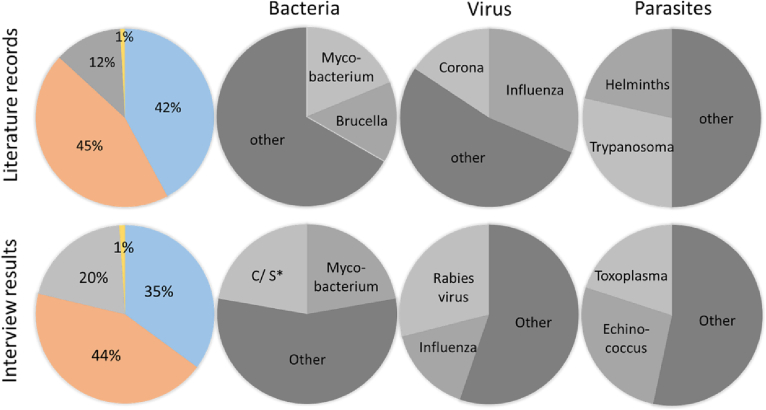
**Relative frequencies of pathogen domains and species based on literature review and experts' interviews.** Relative abundance is shown for literature records (top row) describing bacterial (blue, *n* = 96), viral (orange, *n* = 102), parasitic (grey, *n* = 28), and fungal (yellow, n = 2) pathogens and interview results (bottom row) describing bacterial (blue, *n* = 35), viral (orange, *n* = 44), parasitic (grey, *n* = 20) and fungal (yellow, n = 1) pathogens. Records were further analyzed for their respective pathogen species (grey-shaded pie charts). The divisions of each circle represent the percentages of the quantities of each species within the whole circle. Other = articles describing more than only one pathogen species or articles on pathogen species found less often than the listed ones, *campylobacter/salmonella. (For interpretation of the references to colour in this figure legend, the reader is referred to the web version of this article.)

Total literature results were analyzed to categorize data according to their pathogen specificity. Whereas articles describing bacterial and viral pathogens were found in similar quantity, literature with a focus on parasites was found less often. The least literature was found on zoonotic fungal pathogens. Of note, both bacterial and viral literature results showed a clear domination of one to two species. The majority of the viral results were dominated by records on influenza virus (31%). This was similar for bacterial results with zoonotic *Mycobacterium* (19%) and *Brucella* (15%) as the most frequently retrieved results (see Fig. 2, top row). In addition, 46% of the records on bacterial pathogens were related to antimicrobial resistance (AMR, data not shown).

Interview results from question 2 (see Table S2) were used to identify which pathogen species are of the participants' interests. The general results show that bacteria (35%) and viruses (44%) were described with similar frequency, followed by parasites (20%) and fungi with only 1%. In detail, zoonotic Mycobacterium and Campylobacter spec./ Salmonella spec. accounted for 22% respectively, viral species were dominated by responses on rabies virus (29%), and Echinococcus species were the most frequently mentioned parasitic pathogens (27%) (see Fig. 2, bottom row). Due to the low number of hits on fungal pathogens, they were excluded from further analysis. The representation of fungal pathogens in each of the first pie charts intended only to show that they were mentioned as well.

### 3.3 Keywords used to describe the human-animal-interface

The results of the literature data showed “transmission” and “zoonosis” as the favorite keywords followed by “contact” and “interface”. The interviewees clearly favored “interface” as the most frequently used keyword followed by “contact” (see Table. 1)

### 3.4 Interface categorization according to pathogen species

Literature screening resulted in seven contact interface categories. General results show that the majority of articles described direct contact as a prioritized contact interface for pathogen spillover followed by consumption of animal products and contact with animal products. Results of the pathogen domain-specific interface categories reveal a more diverse pattern. Whereas the results of the bacteria-focused literature show a clear preference for consumption of products of animal origin followed by direct contact, the data on interface types from viral literature is headed by direct contact followed by contact to products of animal origin, in contrast to vector contact and environmental contact from parasitic-specific interfaces. Although not playing the most important role, environmental contact for bacterial pathogens and indirect contact for viral pathogens, those categories were commonly mentioned as well, however, with a lower frequency. Data describing indirect contact was not found for parasitic pathogens (see Table 2, literature columns).

Additionally, responses given by the interviewees regarding the transmission of a certain pathogen (question 2, see Table S2) were analyzed for the mentioned categories. The results show that, in general, the majority of responses associated consumption of animal products, direct contact, and contact with animal products as an interface of their named pathogen species. In more detail, the results of the pathogen domain-specific interface categories revealed a more distinctive pattern. Most of the responses on bacterial and parasitic pathogen species linked the consumption of animal products to pathogen spillover. On the other side, the participants associated pathogen spillover of viral pathogen species more likely with contact with animal products compared to any other interface category (see Table 2, expert columns).

### 3.5 Interface categorization based on an operational approach

**Fig. 3:**
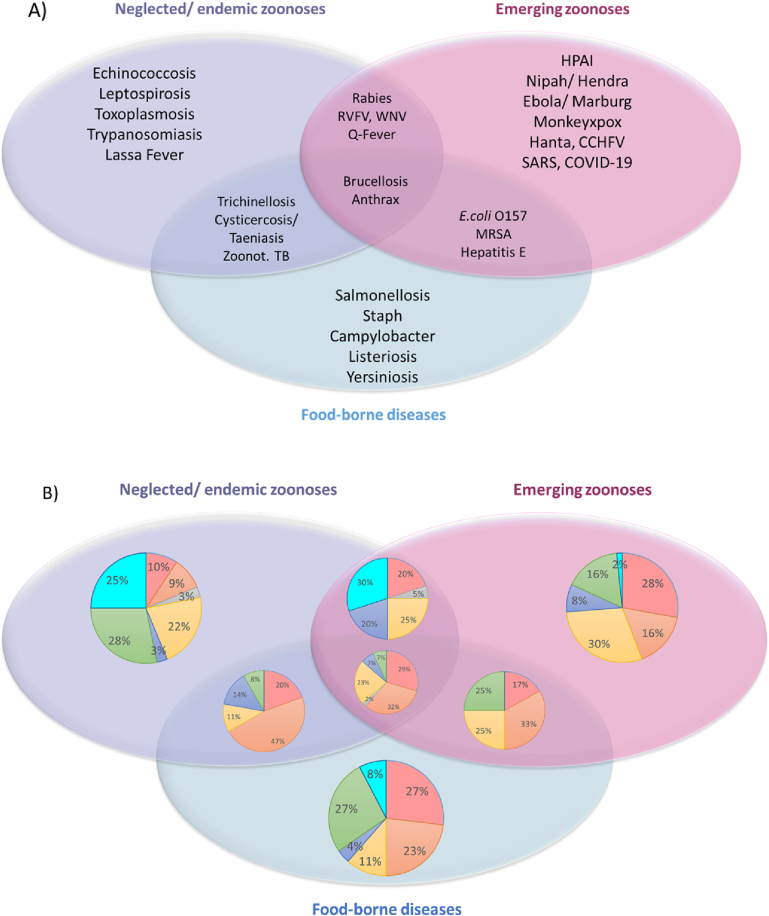
**Categorization of contact interfaces on the basis of the FAO APPROACH TO ZOONOTIC DISEASES.** A) Pathogen species retrieved from the literature search were grouped according to the FAO APPROACH TO ZOONOTIC DISEASES (see Fig. S4). B) Contact interface categories were calculated based on the scheme applied in A. Data are provided in percentages. Legend of the circles: direct contact (), consumption of animal products (), use of animal products (), contact with animal products (), indirect contact (), environmental contact (), vector contact (). Legend to A: CCHFV = Crimean Congo Haemorrhagic Fever virus, TB = Tuberculosis, Staph = Staphylococcus, MRSA = Methicillin-resistant *Staphylococcus aureus*.

To investigate relevant contact interface types from a practical point of view, pathogen-specific data were analyzed based on the “FAO Approach To Zoonotic Diseases” (see Fig. S4). Therefore, a modified scheme, consisting of three main operational areas, was created by including pathogen-specific literature data (see Fig. 3). In addition, diseases, which are associated with two or more main areas, are presented as connecting areas. Based on the data from Fig. 3A, relative frequency of interface categories was calculated and depicted in Fig. 3B. The results show that neglected/endemic zoonoses are mainly driven by environmental contact (28%) followed by vector contact (25%), whereas emerging zoonoses are mainly associated with contact to animal products (30%) and direct contact (28%). Surprisingly, for food-borne diseases, the main interfaces are associated with direct (27%) and environmental contact (27%). For zoonotic diseases located within the connection area between neglected/endemic zoonoses and emerging zoonoses, the most important interface with the transmission of these diseases is associated with vector contact (30%). Consumption of animal products is the most important interface of the connection areas between neglected/endemic zoonoses and food-borne diseases (47%) as well as the connection areas between emerging zoonoses/food-borne diseases (33%) on the one hand and all of them together (32%) on the other hand. It is also noted that vector contacts do not play a role in the pathogen transmission of those.

## 4 Discussion

Although there is no definition available, the term human-animal-interface is widely used in the literature. In this study, the understanding of the term human-animal-interface was investigated based on a systematic literature analysis and from health experts' perspective by identifying keywords and descriptions, transmission pathways, and linked pathogen associated with the term. Most frequently identified keywords varied between literature and experts. Seven contact interface categories were derived showing specific patterns between different pathogen domains, and general concordance between literature analysis and experts' views.

When looking into the dynamics of literature hits over time, we can conclude that overall trends in zoonotic disease perception are reflected in the publishing records. A steady increase in literature using the term “human-animal-interface” was found with a sudden jump in 2020. Here, 30% of the included literature with a virologic focus associated to coronaviruses with articles on COVID-19 accounting for 83%. Although the general increase was no longer apparent in 2021, there was still an upward trend in relevant coronavirus literature with 42% of the virus-related literature, of which COVID-19 articles still accounted for 63%. In contrast, in 2019 no coronavirus-related records were identified for analysis. However, it remains to be mentioned that although COVID-19 had not played a role before 2019, coronavirus literature could be readily found (e.g. Middle East Respiratory Syndrome). On the other hand, the matches for zoonotic influenza among the virus-related articles declined in 2020 (25%) compared to 2019 (60%), which indicates a switch in awareness and priorities. This observation was also true for another public health threat at the human-animal-interface, i.e. antimicrobial resistance (AMR), as the number of records in general with AMR-related content has almost halved in 2020 (19.5%) compared to 2019 (38%). This tendency could also be observed in the bacteria-associated literature with an almost halving of hits in 2020 (41%) compared to 2019 (73%), although the overall number of bacteria-associated records increased.

The literature results showed a clear preference for relating the interface between humans and animals with the terms “transmission” and “zoonosis” followed by “contact” and “interface” itself. While the identification of keywords used to describe the interface or to relate content to the human-animal-interface was rather straight, it was more difficult to identify a categorization of the human-animal-interface. Usually, the transmission is described as being direct or indirect. While the direct interface was defined as physical contact between the species, the indirect interface provides a much broader way of understanding. Therefore, the indirect interface term was further subcategorized into seven subjective interface categories. When comparing the results of the literature between the general interface categories and the pathogen domain-specific interface categories, it seemed that the preference of a certain interface category highly depended on the pathogen domain and the involved pathogens themselves. In addition, the different preferences found between the literature and experts revealed that a pathogen domain-specific pattern could be no more than a snap-shot since it highly depends on the subjective pathogen focus, which is ultimately determined by the subject's area of interest. Expert interviews showed some differences to the literature, but follow a comparable pattern. Although all of them provided a description of their own understanding regarding the human-animal-interface, 96% of them came up with a definition (see Fig. S2A). Further analysis revealed, that neither the pandemic situation, which started after the first interview round, nor the organizational orientation was associated with the availability of a definition (see Fig. S2B-E). Thus, the outbreak of COVID-19 might not have influenced the participants' awareness of that topic.

Interestingly, the analysis of these results revealed more of a tendency towards using “contact” after omitting “interface” as the most frequently used synonym here, which might be biased by being part of the question. The interviewee's own understanding of the interface complicated the categorization of the term. Since the interface categories were already fixed by the analysis of the literature results, the interviewees often had to specify it more precisely. Furthermore, the description of the interface was frequently mixed up with influence factors or drivers such as agriculture or weather conditions. This in turn also indicates how difficult but also subjective it is to define the interface appropriately.

Another approach was to analyze the interface categories using the “FAO Approach To Zoonotic Diseases” as a template (see Fig. S4). This analysis again revealed the dependence of the ranking of each interface category on the pathogen species as well as the description of the interface by the authors. For example, the analysis of the food-borne disease group showed a clear preference for transmission via direct contact, whereas the interface for those pathogens to spill over via consumption ranked second. In contrast, the pathogens causing diseases grouped between neglected/endemic zoonoses and food-borne diseases or grouped between all of them were preferentially transmitted via the consumption of animal products. On the one hand, these findings appeared surprising, on the other hand, they might be well explained through the included pathogen species and the description of the associated interface by the authors. In addition, the general weak data availability for the parasitic domain led to an outnumbering of parasitic pathogen species by viral or bacterial ones. This means, no matter how often a parasitic-caused zoonosis was included in either of these groups, the presence of viral or bacterial pathogen species and their associated interface description resulted in an underrepresentation of the parasitic-related interfaces.

One highly important aspect that had emerged was the almost complete lack of knowledge about the impact of drivers at the human-animal-interface, which enables a spillover and, finally, adaptation to the new host system [15]. While certain influence factors that enhance the probability of pathogen transmission are well known [[16], [17], [18], [19], [20]], studies that quantitatively assess human-made factors are scarce. Especially the wildlife sector seems to be still a red rag in this regard as investigations on environmental conditions in relation to dynamics of hosts or vectors as well as pathogen survival and propagation are limited [21]. It was mentioned that there is increasing concern about managing this human-wildlife-interface in a safe way as we know so little [18]. What is really needed, is to understand the value chains of consuming and trading wild animals and which animal species in particular. The interviewee mentioned that, very often, national controls categorize traded wild animals as such without recording species names which makes it impossible to associate the transmission of pathogens with the particular species. All these aspects are necessary to finally enable an assessment of factors, which support pathogen jump at the human-animal-interface. In addition, a better understanding of the human-animal-interface is relevant for approaches considering human behavior [22]. This applies particularly to countries with high infectious disease burden due to poverty and low social status [[23], [24], [25]]. When you ignore these components in the fight against zoonotic diseases and AMR and especially when it comes to policy, you might lose the game.

### 4.1 Limitations

The interview part of the study was prone to several limitations impeding a well-encompassing reflection of the target population. The recruitment of interview candidates (*n* = 44) was based on existing contacts to experts with mainly veterinary public health backgrounds. -Thus, a veterinary-specific imbalance of opinions cannot be excluded. Most experts work at the same federal research institute (*n* = 16), which might have biased interview results towards a more national and laboratory-based view. Although the response rate (27/44: 61.4%, excl. 6.8% pending) was high, the lack of responses, especially on the international level, might have additionally influenced the outcome of the interview part. The information provided by the participants was based on their expertise in the field of zoonoses/One Health. However, since each participant has his/her own pathogen-restricted specialization, the information given beyond that could be prone to bias. The most important data source was represented by literature records. Although a systematic search in two databases including references of identified records was performed, relevant documents might have been missed. Therefore, it is noted that the number of relevant records was presumably affected by the chosen search strings.

## 5 Conclusion

The term “human-animal-interface” is frequently used in a scientific context. Analyzing the usage of the term in scientific literature reveals, that it remains a wide field of what people try to cover with the term human-animal-interface, depending on pathogen domain, background, and rationale of their respective study. However, despite the lack of a formal definition, the term seems not to be used in contradicting or conflicting ways. From that point of view, striving for a more rigid definition as a standing technical term will not bring additional advantages to the scientific discourse On the other hand, the human-animal-interface is identified as term and thematic area requesting for more knowledge on evolutionary drivers and current developments.

## Funding

This research was partly funded by the Global Health Protection Programme of the German Ministry of Health, grant number ZMVI1-2519GHP701.

## CRediT authorship contribution statement

**S. Dreyer:** Data curation, Formal analysis, Investigation, Visualization, Writing – original draft, Methodology, Writing – review & editing, Resources. **M. Dreier:** Methodology, Writing – review & editing, Supervision. **K. Dietze:** Conceptualization, Project administration, Resources, Supervision.

## Declaration of Competing Interest

The authors have no competing interests to declare.

## Data Availability

Non-confidential data can be shared upon request. Data containing confidential components can be shared anonymized.

## Appendix A Supplementary data

Supplementary material Image 8
