# Supplementary material for: Demystifying a buzzword: Use of the term “human-animal-interface” in One Health oriented research based on a literature review and expert interviews
Source: One Health. 2023 May 11;16:100560. doi: 10.1016/j.onehlt.2023.100560 (PMC10288080; doi:10.1016/j.onehlt.2023.100560)
Supplement: Supplementary file 1 — Supplementary material [file mmc1.docx]

**Appendix A. Supplementary methods, material and results**

**Demystifying a buzzword: Qualitative analysis of the utilization of the term „human-animal-interface“ in One Health oriented research.**

Sylvia Dreyer^a^*; Maren Dreier^b^; Klaas Dietze^a^

^a^ Friedrich- Loeffler Institut, Institute of International Animal Health/One Health, Greifswald, Germany

^b^ Hannover Medical School, Institute for Epidemiology, Social Medicine and Health Systems Research, Hannover, Germany

Corresponding author:

*Sylvia Dreyer, Institute of International Animal Health/ One Health, Südufer 10, 17493 Greifswald- Insel Riems, Germany, [sylvia.dreyer@fli.de](mailto:sylvia.dreyer@fli.de)

**Text S1. Literature search**

**Data collection**

The following search strings were used: human-animal-interface, animal-human-interface, human-livestock-interface, livestock-human-interface, wildlife-human-interface and human-wildlife-interface. All data on relevant articles were collected by the main researcher and digitally stored. Own search strategy was applied. Updates of literature matches were achieved by rerunning searches. Since the search strategy included no sensitive restrictions and was conducted manually, no peer review process was necessary. In total 644 records in total were identified. Deduplication was conducted manually. Therefore, each list of records was printed and duplicates were marked by hand. After deduplication and eligibility screening, 208 records were included in further analysis (details, see Fig. 1A). All findings were stored at the workplace. Printed record lists were stored in folders and records were stored digitally.

**Data analysis**

Excel table included quantitative data on: the organisms and the first author of the study, the type of article, the seven interface categories (see results, Table 2), AMR problems, genetic interface types (recombination, mixing vessel, other), environmental interface (geography and season), if the interface was mentioned at all and how many times, synonyms used and their quantity (see results, Table 1), set-up of the study (methods described, study type, samples taken) and if an outlook was provided. This table was filled by information from each single article. In addition, a word file contained qualitative information in more detail. Since more data was collected as intended for the study, not all data was included in the study.

**Text S2. Expert interviews**

**Study design**

To underpin the study, a content analysis of the interviews was conducted to collect actual and experience-based data. No statistical validation of the interviewees was applied beforehand. The aim of the study was to include as many experts in this field as possible. The procedure of candidate recruitment is visualized in Figure S1.

**Fig. S1.** Procedure of candidate inclusion


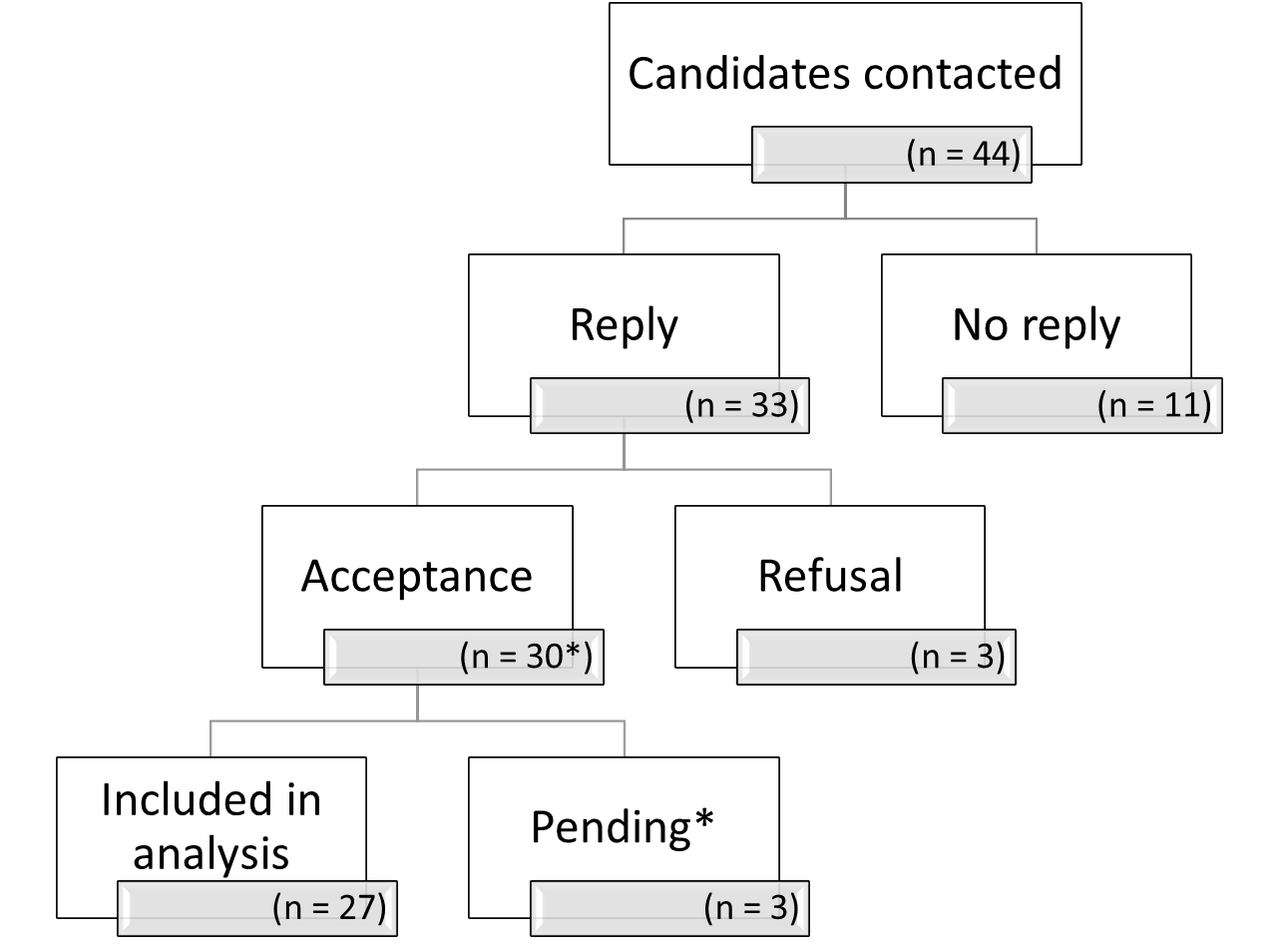


Step by step procedure of the inclusion process of candidates. Legend: refusal = self-declared as not being an expert in this field, *no confirmed transcript received or no transcript sent yet.

**Interview participants and inclusion process**

Participation was voluntary. Prepared privacy forms were sent by e-mail to obtain written consent of each participant and permission to voice-recording of the interviews. The form contained information on the study background and the interview process. Furthermore, information on data acquisition and data protection was provided and the use of pseudonymized personal data. To ensure total confidentiality for the participants, only the main researcher (SD) had access to the recordings and integral transcripts. Due to problems in technical understanding of one transcript, a colleague was asked to conduct a language proof with a pseudonymised voice-record of that interview. All experts working in the field of zoonosis/ One Health were contacted by e-mail by SD. Candidates who accepted participation replied by sending the signed privacy form and suggesting a preferred time slot. In accordance with the candidate, a date was arranged for the zoom interview.

**Table S1a:** General information about the interviewees

| Characteristics |  |
| --- | --- |
| Number of participants | n = 27 |
| Sex | Female: n = 9  Male: n = 18 |
| Place of work | National level: n = 17  International level: n = 10 |
| Professional background | Veterinarian: n = 22  Biologist: n = 3  Medical doctor: n = 1  Public Health: n = 1 |
|  |  |
|  |  |
|  |  |
| First three questions asked | 2019: n = 10  2021: n = 17 |

**Table S1b:** Detailed information on the current area of work of the interviewees

| Professional  background | Current area of work | | | | | |
| --- | --- | --- | --- | --- | --- | --- |
|  | FLI | WHO | FAO | WOAH | CDC | BMZ |
| Veterinarian | 13 | 1 | 5 | 2 | 1 |  |
| Biologist | 3 |  |  |  |  |  |
| Medical doctor |  |  |  |  |  | 1 |
| Public Health |  |  |  | 1 |  |  |

Indicated numbers reflect the numbers of interviewees

**Data collection**

General process. Questions were asked only once and ad hoc and not shared beforehand. The questions were pilot tested with the first two participants, and as no changes were made, they were included in the data set. Interviews at the first time point were conducted face-to-face. The resulting transcript was sent for validation by email. As restrictions due to COVID-19 disabled a second face-to-face interview round, the interview format was changed towards a virtual version. Voice recording was used to collect the data. Interview recordings were transcribed and transcripts returned to the participants for proofreading, validation and signature. All participants validated their version.

Virtual interviews. All zoom interviews lasted 30 minutes each. The interviews were held in German or English. Voice recording was used to collect the data and no notes were made, neither during nor after the interview. Interview recordings were transcribed manually and with regards to content, and the audio recording was stored afterwards at a workplace drive which was access-restricted by the main researcher only. The transcripts were returned to the participants for proofreading, validation and signature. All participants validated their version.

Privacy form. By signing the privacy form, the participants were informed about the data acquisition, meaning: (a) five open-ended questions (see Table S2) needed to be answered which was voice recorded, (b) a short presentation on the results from the literature search was provided, (c) the voice-recordings were transcribed and handed back for consent to the interview partner and (d) the data collection was not a control of legal requirements and data were only used for scientific purposes. Background information about the study was given at the beginning of each interview. For the candidates, who already participated in 2019 two possibilities were offered to answer the two remaining questions, either by email or via zoom interview. Whereas in the first round the questions were asked in German as the participants were FLI colleagues, in the second round the language switched to English since the new participants had an international background. In order to ensure confidentiality to the participants, all identifying information was stored at a drive which was access-restricted by the main researcher only. If relevant, material provided by the candidates was included to improve the study outcome.

**Data analysis**

Five questions were formulated (see Table S2). Interviews were conducted virtually. Interviews were voice-recorded using zoom software (version: 5.6.6) and stored as M4A audio file. Response analysis was facilitated by screening the whole answer (context unit) of each participant (analysis unit) and extracting the relevant information (coding unit) in an Excel table. Relevant information was not identified in advance; it was inductively derived from the data (addition of value). Data extraction was conducted as follows: (Q1) extraction of information for yes/ no answers and synonyms used, (Q2) extracting information on pathogen species/ domains and their associated interface category, (Q3+Q4+Q5) extracting information for yes/ no answers and (Q5) extracting information on the connection between AMR positive responses and their pathogen-domain connection. Variations in theme by age, gender, experience, and qualifications were not examined. Results were checked by SD only. All analysis was carried out on the original English versions. No important aspects of the study and/or those directly related to the objective of the study have been omitted. Participant’s quotations were included anonymously. The procedure applied here is summarized in Figure S3.

**Table S2.**

| Order | Question |
| --- | --- |
| 1 | How would you define the interface between human and animal? Or: What does interface mean to you? |
| 2 | Please name the associated interface based on specific zoonotic pathogens. |
| 3 | From your perspective, are there any open research questions to define the interface? (for known pathogens)? |
| 4 | From your perspective, are there still some unknown interfaces (not yet discovered)? |
| 5 | When you think of the pathogens and their interface mentioned above, where do you see problems specifically related to antimicrobial resistance (AMR)? Or are there any problems at all? |

**Text S3. Availability of a definition for interface in terms of species-crossing pathogens**

Interview responses regarding a definition availability: The first questions aimed to investigate, if the participants had a definition for the human-animal-interface and how they described it. The availability of own definitions was analysed by affiliating the content of the answer either to the presence (yes) or absence (no) of it (see Fig. S2). Whereas most of the participants readily provided a definition (96%), a minority (4%) did not (see Fig. S2A). To acquire more data from the general results in Fig. S2A, the data were analysed further and stratified by year and by institutional level (see Figs. S2B-E). The data show that all participants who answered the first questions in 2019 provided a definition in contrast to those who were interviewed in 2021. A difference was detectable depending on the institutional level as well, where all participants from international organizations had their own definition ready in contrast to those working on national level with 7% lack of a definition. However, none of the differences was significantly associated with year (p 0.47) or institutional level (p > 0.9999).

**Fig. S2.** Defining the interface by interviewees


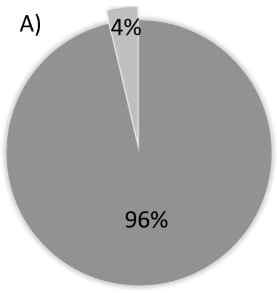

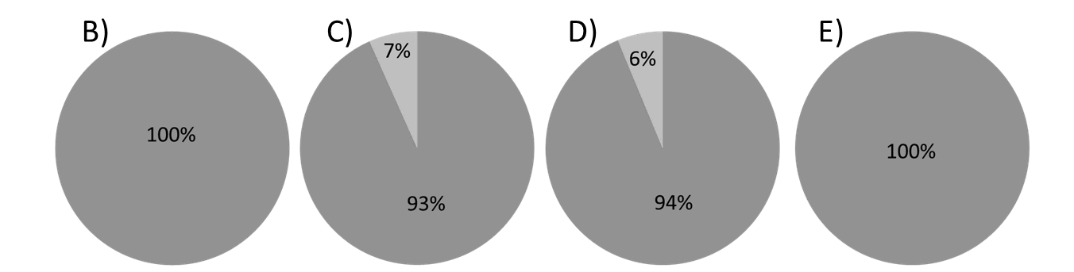


A) Interview responses were grouped into the absence or presence of a definition (n = 27). Results from A were stratified by the year the participants were asked such as B) 2019 (n = 12) or C) 2021 (n = 15) and by the institutional level such as D) national or E) international. The relative frequency of answers is depicted as pie chart. Presence of a definition is shown in dark grey. Absence of a definition is shown in light grey.

**Fig. S3. Procedure of data acquisition from interviews**


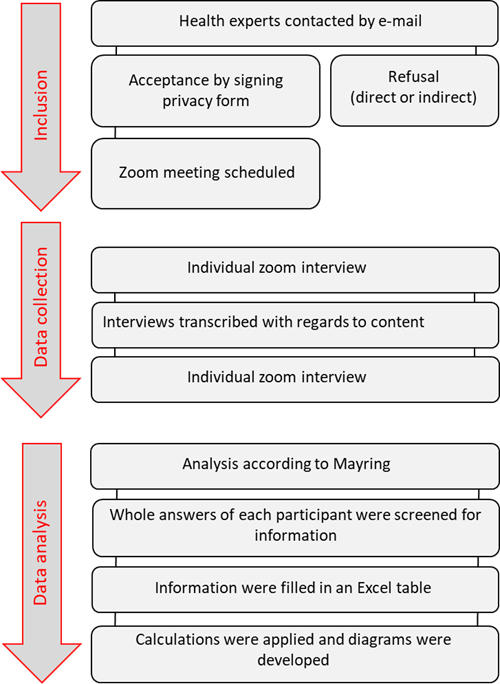


General interview procedure (exemplary for the virtual variant). Experts in the field of zoonosis and/ or One Health were contacted by email and asked to participate. Questions were asked face-to-face in 2019 and via zoom/ email in 2021. A transcript of the interview was provided and sent back for confirmation. Confirmed transcripts were included in the analysis.

**Fig. S4. Categorization of contact interfaces by applying an operational approach.**


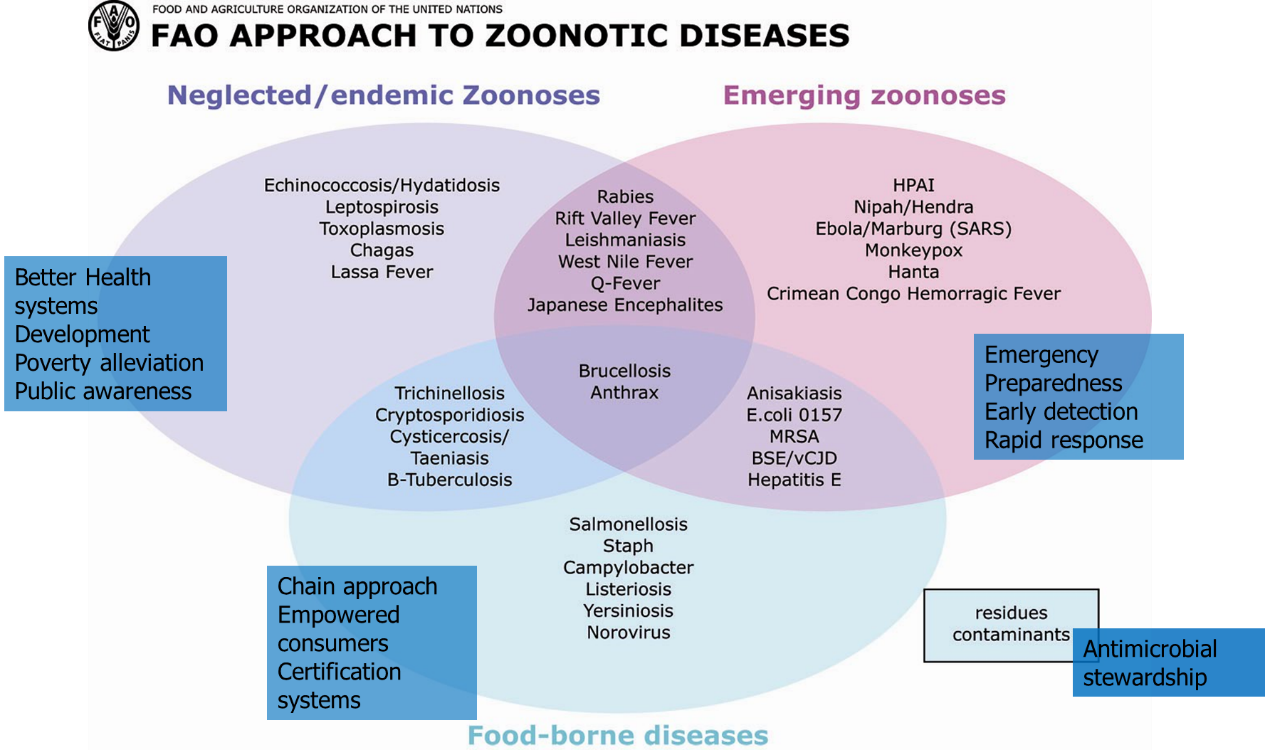


Template of the FAO APPROACH TO ZOONOTIC DISEASES (kindly provided by an interviewee).

Statistics

Stratified results (see Text S2) were statistically analysed using Fisher’s exact test. GraphPad Prism 9 software was used for calculation (Version 9.0.0).
